# Sleep deprivation affects pain sensitivity by increasing oxidative stress and apoptosis in the medial prefrontal cortex of rats via the HDAC2-NRF2 pathway

**DOI:** 10.1016/j.bj.2024.100826

**Published:** 2025-01-02

**Authors:** Shuhan Chen, Yanle Xie, Zenghui Liang, Jing Liu, Jingping Wang, Yuanyuan Mao, Fei Xing, Xin Wei, Zhongyu Wang, Jianjun Yang, Jingjing Yuan

**Affiliations:** a Department of Anesthesiology, Pain and Perioperative Medicine, The First Affiliated Hospital of Zhengzhou University, No. 1 Jianshe East Road, Zhengzhou, Henan Province, China; b Henan Province International Joint Laboratory of Pain, Cognition and Emotion, Zhengzhou, Henan Province, China; c Massachusetts General Hospital Department of Anesthesia, Critical Care and Pain Medicine, Harvard Medical School, Boston, MA, USA

**Keywords:** Sleep deprivation, Pain, Oxidative stress, HDAC, NRF2

## Abstract

Sleep is crucial for sustaining normal physiological functions, and sleep deprivation has been associated with increased pain sensitivity. The histone deacetylases (HDACs) are known to significantly regulate in regulating neuropathic pain, but their involvement in nociceptive hypersensitivity during sleep deprivation is still not fully understood. Utilizing a modified multi-platform water environment technique to establish a sleep deprivation model. We measured the expression levels of HDAC1/2 in the medial prefrontal cortex (mPFC) through immunoblotting and real-time quantitative PCR. The presence of pyroptosis was determined using a TUNEL assay. Suberoylanilide hydroxamic acid (SAHA), an HDAC inhibitor employed clinically, was injected into the peritoneal cavity to inhibit HDAC2 expression. Animal pain behaviors were evaluated by measuring paw withdrawal thresholds (PWTs) and paw withdrawal latencies (PWLs). Our findings indicate that sleep deprivation leads to increased nociceptive hypersensitivity, an upregulation of HDAC2 expression in the mPFC, a downregulation of the expression of nuclear factor erythroid 2-related factor 2 (NRF2), and changes in markers of oxidative stress in rats. SAHA, the HDAC inhibitor, enhanced NRF2 expression by inhibiting HDAC2, which consequently ameliorated oxidative stress and mitigated nociceptive hypersensitivity in rats. The incidence of apoptosis was found to be higher in the mPFC tissues of sleep deprivation rats, and the intraperitoneal administration of SAHA decreased this apoptosis. The co-injection of SAHA and the NRF2 inhibitor ML385 into sleep deprivation rats negated the beneficial effects of SAHA. In conclusion, HDAC2 is implicated in the induction of oxidative stress and apoptosis by suppressing NRF2 levels, thereby exacerbating nociceptive hypersensitivity in sleep deprivation rats.

## 1 Introduction

Sleep is a complex and essential physiological process for the human body. Both acute and chronic sleep insufficiency can have adverse effects on its functioning. Sleep deprivation occurs due to inadequate sleep quantity or quality, leading to a chronic state where partial sleep restriction accumulates over days, weeks, or months, resulting in sleep debt. Studies have consistently shown the numerous negative impacts of sleep deprivation on the body, emphasizing the link between sleep and pain [1,2], as chronic pain conditions are frequently associated with sleep deprivation.

In recent years, a multitude of experimental studies have been conducted to evaluate the impact of sleep deprivation on pain [3]. These studies have consistently reported an increase in nociceptive hypersensitivity associated with sleep deprivation. Although our comprehension of the link between sleep and pain has improved, the underlying mechanisms of pain following sleep deprivation are still not fully understood. There is an immediate need to elucidate these mechanisms and devise effective therapeutic strategies for this persistent problem that has affected people for many years.

Histone deacetylases (HDACs) are instrumental in facilitating chromatin compaction and silencing gene transcription by removing acetyl groups from histone tails [4]. Their modulatory influence on diverse forms of pain has been well-documented, with corresponding analgesic effects observed for their inhibitors [5,6]. Among the classical class I HDACs, HDAC2 has been implicated in neuropathic pain, inflammatory pain, and bone cancer pain [[7], [8], [9]].

The medial prefrontal cortex (mPFC) is pivotal in the processing of various types of pain, with observable circuitry changes evident in models of neuropathic pain [10]. In rodent models of chronic pain, substantial evidence indicates mPFC dysfunction, which is associated with structural and functional changes in pyramidal neurons [11,12]. Notably, both chronic postoperative pain and stress-induced pain responses have been shown to elicit alterations in HDACs within the mPFC [13,14]. The involvement of HDACs in mPFC-mediated sleep deprivation-induced pain and the mechanisms at play are not yet fully understood.

Previous research from our laboratory has indicated that a single night of sleep deprivation in healthy individuals results in reduced pain thresholds for pressure and changes in blood markers indicative of oxidative stress [15]. Oxidative stress leads to neural damage and severe neuropathy [16]. Further studies in experimental animals have linked oxidative stress during sleep deprivation to an increased sensitivity to pain, known as hyperalgesia [17,18]. The nuclear factor erythroid 2-related factor 2 (NRF2), a transcription factor sensitive to redox changes, is typically found in the cytoplasm in conjunction with the Kelch-like ECH-associated protein 1 (Keap1) under normal physiological conditions. When oxidative stress occurs, NRF2 detaches from the Keap1-NRF2 complex, moves to the nucleus, and offers protection against oxidative stress by modulating the expression of endogenous antioxidants, phase II detoxification enzymes, and other cellular defense mechanisms through antioxidant response elements (ARE) [19]. Additionally, NRF2 modulates the expression of anti-apoptotic proteins, including Bcl-2 [20].

Additional studies have shown that activating NRF2 mitigates cognitive impairment in mice caused by sleep fragmentation by reducing neuronal apoptosis in the hippocampus [21]. Extensive research has corroborated that histone deacetylases (HDACs), particularly HDAC1 and HDAC2, play a regulatory role in NRF2 [22,23]. Apart from oxidative stress, sleep deprivation is also likely to influence neuronal apoptosis. Investigations have uncovered that sleep deprivation can precipitate neuronal loss by triggering the pro-apoptotic protein Bax, activating caspase-3 and autophagy-related proteins, and simultaneously lowering the levels of the anti-apoptotic protein Bcl2 [24]. In rats experiencing chronic pain, two consecutive weeks of sleep deprivation have been shown to escalate the incidence of neuronal apoptosis within the hippocampus [25].

Therefore, our hypothesis suggests that HDACs may contribute to the pain associated with sleep deprivation. We anticipate that the inhibition of HDACs could potentially alter the body's pain sensitivity via the NRF2 pathway, which is involved in the regulation of oxidative stress.

To test our hypothesis, we established a rat sleep deprivation model. We compared the expressions of HDACs (HDAC1 and HDAC2) in the mPFC of sleep deprivation rats to assess the involvement of HDACs in the sleep deprivation process. We selected suberoylanilide hydroxamic acid (SAHA), an HDAC inhibitor approved by the Food and Drug Administration, for intraperitoneal administration to the rats. This strategy was designed to explore if the inhibition of HDACs by SAHA could alleviate oxidative stress via the NRF2 pathway, thereby potentially reducing the pain associated with sleep deprivation.

## 2 Method

### 2.1 Animals

This research was approved by the Ethics Committee of Zhengzhou University. Adult male Sprague-Dawley rats, sourced from SPF (Beijing) Biotechnology Co., Ltd., weighing 200∼220 g, were housed in groups of 6 per cage under a 12-h light/dark cycle, with lights on at 7:00 a.m. The temperature was maintained at 23 ± 1 °C, and both food and water were available to the animals ad libitum. Prior to the experiment, all animals were allowed to acclimate to the experimental conditions for a period of 7 days. The experimental animals were randomly assigned into 9 groups, ensuring that animals within each group were matched for age and weight.

### 2.2 Sleep deprivation protocol

Rats were divided into two groups: the control group (Sham) and the sleep deprivation group (SD). This study employed chronic sleep deprivation, involving several hours (typically 3–8 h per day) of sleep deprivation daily within a 24-h sleep-wake cycle [26]. Previous research has found that chronic sleep deprivation of 6 h per day can exacerbate pain behavior in rats with chronic pain [25]. Therefore, this study involved depriving the rats of sleep for 6 h each day, from 9:00 a.m. to 3:00 p.m. During the sleep deprivation period, pain behavior assessments were conducted every two days to monitor the development of pain hypersensitivity. The study found that rats developed pain hypersensitivity behavior on the 6th day of sleep deprivation, hence the SD group was subjected to 6 days of sleep deprivation, with 6 h per day. After completing the hyperalgesia test and being identified with increased pain sensitivity, the SD group was allowed to recover sleep unrestrictedly in their home cages, while their pain thresholds were monitored to observe their recovery time. Meanwhile, the Sham group was also allowed unrestricted sleep in their home cages, with both groups kept in the same room. Rats designated for drug administration underwent sleep deprivation immediately following the drug injection.

### 2.3 Sleep deprivation model

The best and most common model reported in the literature is the modified multi-platform technique. In this study, animals in the cage (4–6 rats) are placed in a water-filled tank (123 × 44 × 44 cm) at room temperature. The tank has 18 platforms, each about 1 cm above the water surface, with a diameter of 6.5 cm, allowing rats to freely move from one platform to another [27]. When animals reached the paradoxical phase of sleep (REM) sleep, its inherent muscle paralysis causes the rat to come into contact with the water and wake up, thereby completing sleep deprivation. Before sleep deprivation, the rats were trained for 3 days (1 h per day). During the experiment, rats have free access to fresh water and food pellets.

### 2.4 Drugs, and drug administration

The two inhibitors utilized in our study, the HDAC inhibitor (SAHA) and the NRF2 inhibitor (ML385), were procured from MCE (Med Chem Express, Monmouth Junction, New Jersey, USA). The dosages of the drugs were selected based on prior research and our preliminary trials. To examine the regulatory influence of HDAC1/2, SAHA was administered to inhibit the expression of HDAC1/2 in rats, following the method detailed in a previous publication [28]. The rats received an intraperitoneal injection of SAHA at a dosage of 20 mg/kg, prepared in saline with 5% dimethyl sulfoxide (DMSO, Beijing Solarbio Science & Technology Co., Ltd), for a period of 6 consecutive days. The analgesic effects of both SAHA and ML385 were recorded. For assessing the regulatory impact of NRF2, ML385 was administered to inhibit NRF2 expression in rats, as per a previously reported method [29]. The rats were given an intraperitoneal injection of ML385 at a dosage of 30 mg/kg, also prepared in saline with 5% DMSO, daily for 6 days. Western blotting was employed to verify the inhibition of HDAC1/2 or NRF2. On the seventh day of sleep deprivation, the rats that were intraperitoneally injected were sacrificed for quantitative real-time PCR (qRT-PCR) and protein analysis.

### 2.5 Paw withdrawal threshold

Rats were housed in cages with wire mesh bottoms and allowed to acclimate for 30 min before the commencement of testing. We employed von Frey monofilaments, ranging from 2.0 to 26.0 g in force (2, 4, 6, 8, 10, 15, and 26 g; Stoelting, Wood Dale, IL, United States). These filaments were applied perpendicularly to the plantar surface of the paw with a force sufficient to elicit a slight paw flexion. A paw retraction, withdrawal, or licking was regarded as a positive response. Following a positive response, a monofilament with a lower force was used. Conversely, in the absence of a response, a stiffer filament was applied. The "up and down" method was employed to ascertain the paw withdrawal threshold.

### 2.6 Paw withdrawal latency

The Model 336 Analgesic Instrument (IITC Inc. Life Science Instruments, Woodland Hills, CA, United States), was utilized to assess the paw withdrawal latencies (PWLs) in response to noxious heat stimuli. Rats were positioned on a glass platform with a designated testing area and allowed to acclimate for a duration of 30 min before the procedure commenced. The radiant heat stimulator was directed at the plantar surface of the hind paw via a glass plate. A characteristic response, such as the lifting or licking of the hind paw, marked the nociceptive endpoint of the radiant heat test, with the time to this endpoint being recorded as the PWL. To prevent potential tissue damage, a cut-off time of 15.1 s was implemented. Each animal underwent five trials, with a 10-min interval between trials for each side.

### 2.7 Western blot assay

After anesthesia, the rats were euthanized, and their brains were carefully dissected on ice to extract the medial prefrontal cortex (mPFC). The excised tissues were immediately plunged into liquid nitrogen for rapid freezing and subsequently stored at −80 °C for later use. Total protein was extracted from the harvested specimens using a lysis buffer (Beijing Solarbio Science & Technology Co., Ltd), which included a cocktail of protease inhibitors. The protein concentration of the samples was quantified using the bicinchoninic acid (BCA, Beijing Solarbio Science & Technology Co., Ltd) assay. The protein samples were prepared for Western blot analysis, subjected to denaturation at 100 °C for 5 min, and then separated by gel electrophoresis on 10% SDS-polyacrylamide gels. Following electrophoresis, the proteins were transferred onto polyvinylidene difluoride (PVDF) membranes at a current of 200 mA for 1.5 h using a transfer apparatus from Millipore, Billerica, MA, USA. The membranes were then pre-incubated for 1 h in Tris-buffered saline with 0.02% Tween-20 (TBS-T) and 3% non-fat milk, followed by overnight incubation with the respective primary antibodies at 4 °C with gentle agitation. The primary antibodies used were rabbit anti-HDAC1 (1:10000), rabbit anti-HDAC2 (1:5000), and rabbit anti-NRF2 (1:5000), all sourced from Proteintech (Wuhan, China), as well as rabbit anti-β-actin (1:3000). The secondary detection was performed using horseradish peroxidase-conjugated anti-rabbit IgG (1:5000; Proteintech). The immunoreactive bands were visualized using a highly sensitive enhanced chemiluminescence (ECL) detection kit (Biosharp, Hefei, China). The immunoblots were then analyzed using a Molecular Imager (ChemiDoc XRS; Bio-Rad, CA, USA) and quantified with the ImageJ software (National Institutes of Health, Maryland, USA).

### 2.8 Real-time quantitative PCR

Total RNA was extracted from the mPFC tissues using a total RNA extraction kit (Catalog Number DP419, TIANGEN Biochemical Technology Co., Ltd., Beijing, China) following the manufacturer's protocol. The PrimeScript RT Master Mix (Catalog Number RR047A, TaKaRa, Dalian, China) was utilized for the reverse transcription of RNA. Target genes were then amplified in triplicate using the TB Green Premix Ex Taq (Catalog Number RR420A, TaKaRa, Dalian, China) on an Applied Biosystems StepOnePlus Real-Time PCR System (Foster City, CA, United States). The primer sequences used in the experiment are listed in [Table 1]. The relative expression of the target genes was calculated using the 2^−ΔΔCt^ method and normalized to the reference gene GAPDH.

**Table 1:** Sequences of primers used.

Gene	Primer	Sequence
HDAC1	Forward	GTGGCCCTGGACACAGAGAT
HDAC1	Reverse	GCTTGAAATCTGGTCCAAAGT
HDAC2	Forward	CAACCTAACTGTCAAAGGTCACGC
HDAC2	Reverse	TGAAGTCTGGTCCAAAATACTCGA
Nrf2	Forward	GAATAAAGTTGCCGCTCAGAA
Nrf2	Reverse	AAGGTTTCCCATCCTCATCAC
GAPDH	Forward	GAAGGTCGGTGTGAACGGAT
GAPDH	Reverse	CCCATTTGATGTTAGCGGGAT

### 2.9 Immunofluorescence

The rats were anesthetized, and then the tissues were fixed by perfusion with a 4% paraformaldehyde solution. The brain tissues were subsequently extracted and immersed in the same paraformaldehyde solution before being processed for paraffin embedding. Following this, the paraffin-embedded sections were deparaffinized, rehydrated, and washed with phosphate-buffered saline (PBS) solution at room temperature. Antigen retrieval was conducted using a Tris/EDTA buffer at pH 9.0. Afterward, the sections were rinsed with PBS three times and incubated with 3% hydrogen peroxide (H2O2) in methanol for 15 min to block endogenous peroxidase activity. Immunofluorescence staining was then carried out using antibodies against HDAC2, NRF2, NeuN, GFAP, and Iba1.

### 2.10 Biochemical analysis of oxidative stress

Total protein was extracted from the samples using a lysis buffer that contained protease inhibitors, following the procedure outlined for Western Blot analysis. The concentration of total protein in the samples was then quantified using the BCA method. Subsequently, the following parameters were measured: MDA levels, catalase (CAT) activity, and total superoxide dismutase (SOD) activity.

Tissue MDA levels were assessed using a Lipid Peroxidation MDA Assay Kit from Beyotime (Shanghai, China). This method involves a colorimetric reaction where MDA reacts with thiobarbituric acid (TBA) to form a red product, allowing for the quantitative detection of MDA in tissues or lysates. The MDA-TBA complex has a maximum absorption at 535 nm.

Total SOD activity was measured using the Total Superoxide Dismutase Assay Kit with WST-8 from Beyotime. This colorimetric method estimates SOD activity in a sample based on the reduction of WST-8. The reaction yields a stable water-soluble product, and SOD activity is determined by measuring absorbance at a single time point.

Catalase activity in tissues was evaluated using a Catalase Assay Kit from Beyotime. The assay quantifies the peroxidase-catalyzed conversion of hydrogen peroxide chromogenic substrates into red products, which are then measured colorimetrically at 520 nm.

### 2.11 TdT-mediated dUTP nick-end labeling (TUNEL) staining

TUNEL staining was performed using the In Situ Cell Death Detection Kit from Beyotime (Shanghai, China). The mPFC tissues of rats underwent a series of histological processing steps including dehydration, clarification, wax dipping, embedding, sectioning, and baking, followed by dewaxing and rinsing. Proteinase K was applied to the sections, which were then incubated for 20 min at room temperature. After rinsing with deionized water, the sections were placed in a humidified chamber to maintain moisture. Subsequently, 100 μl of 1 × Equilibration Buffer was added to each section and incubated for 15 min at room temperature. TdT Incubation Buffer was added, and a coverslip was placed to ensure even distribution of the reagents. The slides were incubated in a humidified chamber at 37 °C for 60 min, then washed with PBS for 5 min three times. The sections were counterstained with DAPI by dropwise addition, incubated for 5 min in the dark, and then washed four times with PBST solution for 5 min each. Finally, the excess liquid was blotted with absorbent paper, and the sections were mounted with an anti-fade mounting medium and observed under a fluorescence microscope to capture images.

### 2.12 Statistical analysis

Means ± SEM were used to represent data that were normally distributed. The data were analyzed using an unpaired *t*-test for comparisons between two groups and one-way ANOVA for comparisons among more than two groups. PWTs and PWLs were analyzed using two-way ANOVA. When two-way ANOVA indicated significant differences, pairwise comparisons of means were conducted using Sidak's multiple comparison test. All reported *P*-values were two-tailed, with a predetermined significance level of *P* < 0.05. Data analysis and graphing were conducted using GraphPad Prism 8 software (GraphPad, San Diego, CA, United States).

## 3 Result

### 3.1 Sleep deprivation induces mechanical and thermal allodynia

**Fig. 1:**
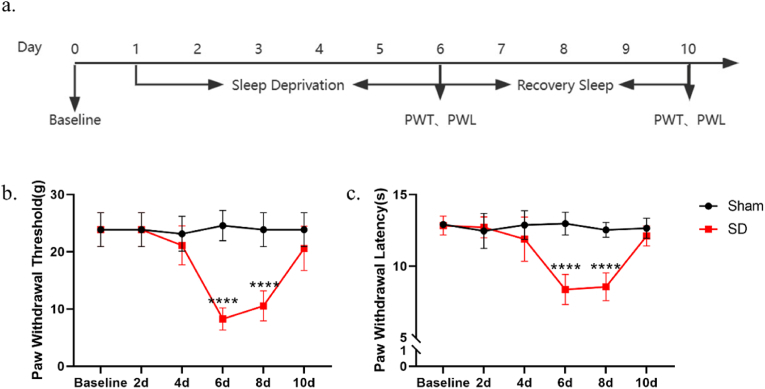
Sleep deprivation causes nociceptive sensitization in rats. (a) The schematic diagram of the experimental arrangement. (PWT: Paw Withdrawal Threshold; PWL: Paw withdrawal Latency). (b) The paw withdrawal threshold of rats at 0,2,4,6,8 and 10 days after sleep deprivation (PWT, *n* = 8, *∗∗∗∗p* < 0.0001 *vs. Sham*). (c) The paw withdrawal latency of rats at 0,2,4,6,8 and 10 days after sleep deprivation (PWL, *n* = 8, *∗∗∗∗p* < 0.0001 *vs. Sham*).

We evaluated hyperalgesia in rats subjected to sleep deprivation by measuring PWTs and PWLs at 2, 4, 6, 8, and 10 days following the initiation of sleep deprivation. The behavioral pain tests indicated that both PWTs and PWLs in sleep deprivation rats significantly decreased after 6 days and returned to baseline levels 4 days post-deprivation cessation [Fig. 1b and c]. The findings indicate that 6 days of sleep deprivation can lead to mechanical and thermal hyperalgesia in rats, whereas 2 or 4 days did not have a significant effect. The results suggest that it takes an accumulative effect of chronic sleep deprivation until day 6 to elicit hyperalgesia. Once hyperalgesia was confirmed in the rats, sleep deprivation was terminated, and PWTs and PWLs were measured every other day. By the 10th day, the rats' pain sensation had returned to normal levels. This indicates that the hyperalgesia induced by 6 days of chronic sleep deprivation requires 4 days of recovery sleep to normalize.

### 3.2 Sleep deprivation induces upregulation of HDAC2 expression and alters oxidative stress markers in the mPFC of rats

**Fig. 2:**
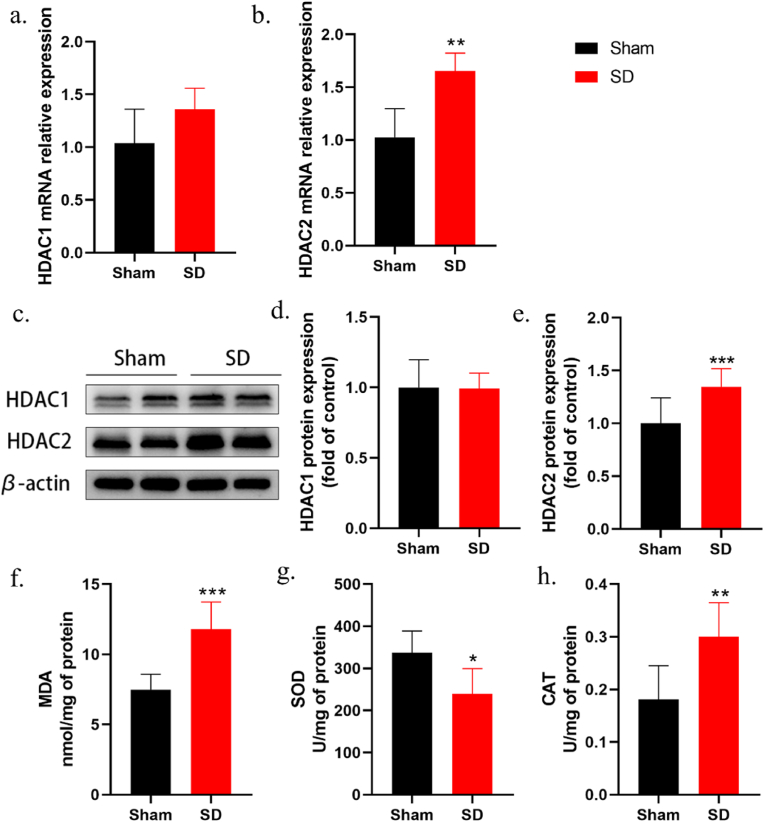
Changes of HDAC1/2 mRNA and protein expression in the mPFC of sleep deprivation rats. (a, b) qRT-PCR results showed that the level of HDAC2 mRNA was significantly increased in the mPFC of rats in the SD group compared with the Sham group, while the level of HDAC1 mRNA did not change significantly (*n* = 4, *∗∗p* < 0.01 *vs. sham*). (c–e) Western blot results showed a significant increase in HDAC2 protein expression, and HDAC1 protein expression levels did not change significantly between the two groups (*n* = 4, *∗∗∗p* < 0.001 *vs. sham*). (f–h) Changes of MDA level, SOD activity and CAT activity in mPFC of rats after sleep deprivation (*n* = 6, *∗p* < 0.05, *∗∗p* < 0.01, *∗∗∗p* < 0.001 *vs. sham*).

To investigate the role of HDACs in central sensitization related to sleep deprivation pathogenesis, we assessed the expression levels of HDAC1 and HDAC2 in the mPFC. qPCR analysis revealed a significant increase in HDAC2 mRNA expression in the mPFC following sleep deprivation, whereas the mRNA expression of HDAC1 remained unchanged [Fig. 2a and b]. In alignment with the qPCR findings, Western blot analysis indicated that sleep deprivation significantly increased HDAC2 protein levels in the mPFC, while the levels of HDAC1 were not significantly altered [Fig. 2c–e]. Thus, we hypothesize that HDAC2 may be involved in the sleep deprivation process in rats.

Following sleep deprivation, the oxidative stress levels in the mPFC of rats were significantly altered. Sleep deprivation resulted in elevated levels of MDA and CAT activity, as well as reduced SOD activity in the mPFC, suggesting an imbalance in redox homeostasis within the tissue [Fig. 2f–h].

**Fig. 3:**
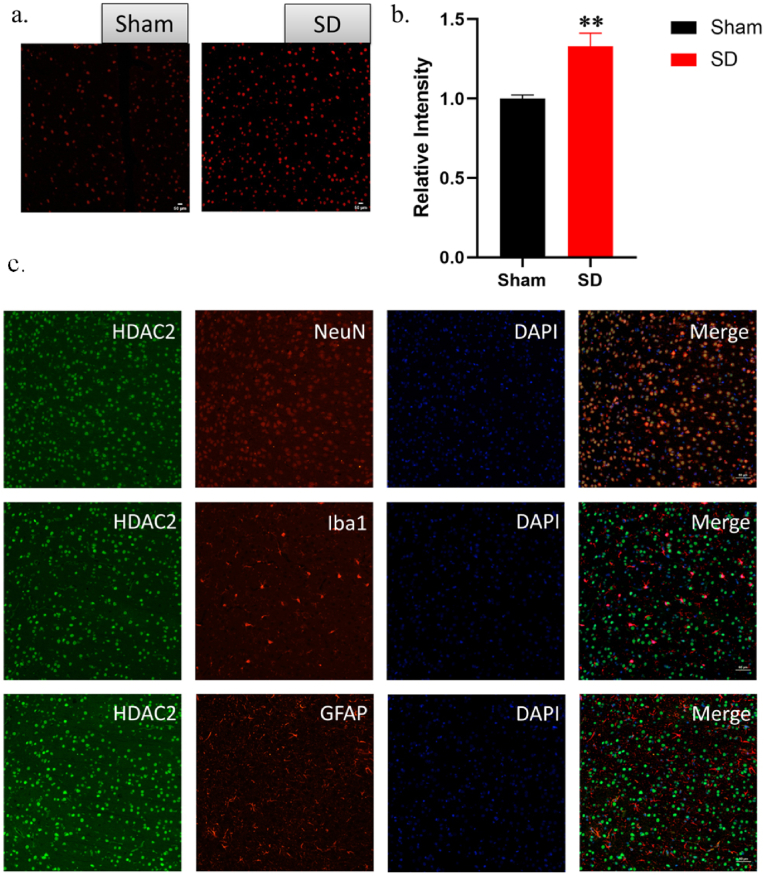
Cellular expression of HDAC2 in the mPFC of rats. (a,b) Immunofluorescence reactivity results of HDAC2 in the mPFC of rats after 6 days of sleep deprivation (*n* = 3, *∗∗p* < 0.01 *vs. sham*). c. Immunofluorescence co-staining of HDAC2 (green) and NeuN (a neuronal marker, red), Iba1 (a microglial cell marker, red), or GFAP (an astrocyte marker, red) in the mPFC of rats. Scale bar, 50 μm. HDAC2, Histone deacetylase 2.

Using immunofluorescence, we detected the expression of HDAC2 in the mPFC of rats. After 6 days of sleep deprivation, HDAC2 immunoreactivity was increased in the mPFC of the SD group compared to the Sham group [Fig. 3a and b]. This evidence suggests that sleep deprivation can upregulate both mRNA and protein levels of HDAC2 in the mPFC of rats. Immunofluorescence double staining indicated that HDAC2 expression was predominantly in neurons (identified by neuronal nuclear antigen, NeuN), with minimal expression in astrocytes (identified by glial fibrillary acidic protein, GFAP) and microglia (identified by the microglial cell-specific marker, Iba1) [Fig. 3c].

### 3.3 SAHA ameliorates sleep deprivation-induced nociceptive hypersensitivity and inhibits HDAC2 expression

**Fig. 4:**
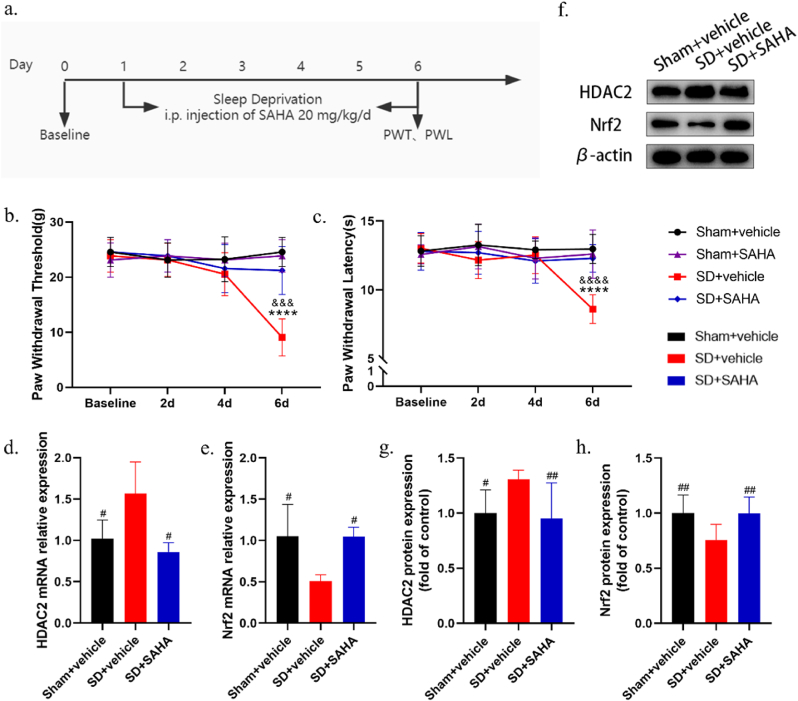
Intraperitoneal injection of SAHA reduces pain sensitivity and inhibits HDAC2 expression in the mPFC of rats. (a) The schematic diagram of the experimental arrangement. (b,c) Effects of intraperitoneal injection of SAHA on PWT and PWL in rats during sleep deprivation (*n* = 8, *∗∗∗∗p* < 0.0001 *vs. Sham + vehicle*; ^*&&&*^*p* < 0.001, ^*&&&&*^*p* < 0.0001 *vs. SD + SAHA*). (d, e) qRT-PCR results showed that the HDAC2 mRNA expression decreased and the NRF2 mRNA expression increased in the SD + SAHA group compared with the SD + vehicle group (*n* = 4, ^*#*^^*p*^ < 0.05 *vs. SD + vehicle*). (f–h) Western blot results showed that the expression of HDAC2 proteins was decreased and NRF2 expression was elevated in the mPFC of rats in the SD + SAHA group compared to the SD + vehicle group (*n* = 4, ^*#*^*p* < 0.05,^*##*^*p* < 0.01 *vs. SD + vehicle*).

To determine whether inhibiting HDAC2 could ameliorate sleep deprivation-induced pain, SAHA, a widely used HDAC inhibitor, was administered intraperitoneally at a dose of 20 mg/kg for 6 consecutive days, beginning on the first day of sleep deprivation [Fig. 4a]. The intraperitoneal administration of SAHA significantly increased PWTs and PWLs in sleep deprivation rats compared to the SD + vehicle group. Moreover, there was no significant difference in PWTs and PWLs between the Sham + vehicle group and the Sham + SAHA group [Fig. 4b and c].

qPCR analysis indicated that intraperitoneal injection of SAHA at 20 mg/kg significantly suppressed the upregulation of HDAC2 mRNA in the mPFC induced by sleep deprivation [Fig. 4d and e]. Western blot results corroborated the qPCR findings [Fig. 4f–h]. Collectively, these results suggest that SAHA treatment may mitigate sleep deprivation-induced mechanical and thermal hypersensitivity, potentially through the inhibition of HDAC2.

Recognizing NRF2 as a key transcription factor in pathways regulating oxidative stress, we further investigated whether SAHA administration attenuated sleep deprivation-induced nociceptive hypersensitivity by reversing the sleep deprivation-induced decrease in NRF2 expression. Western blot and qPCR analyses demonstrated that intraperitoneal injection of SAHA at 20 mg/kg reversed the reduction in NRF2 expression caused by sleep deprivation compared to the SD + vehicle group.

**Fig. 5:**
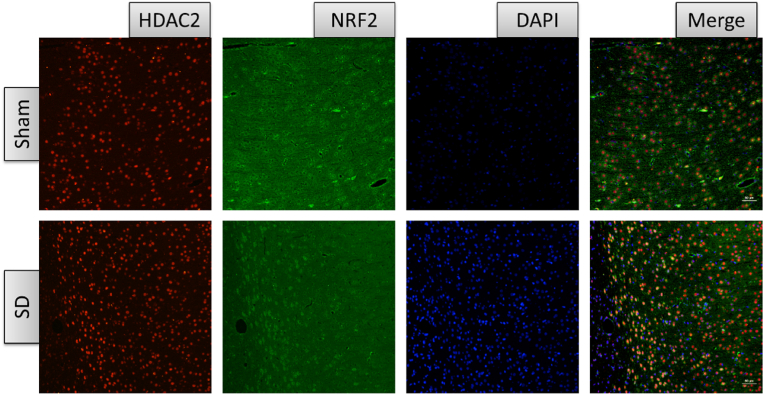
Cellular localization of HDAC2 and NRF2 in the mPFC of rats. Representative images of immunofluorescence co-labeling of HDAC2 and NRF2 proteins in the mPFC of rats from Sham and SD groups. Abbreviations: HDAC2: histone deacetylase 2; NRF2: nuclear factor erythroid 2-related factor 2. Scale bar: 50 μm.

To elucidate how HDAC2 regulates oxidative stress pathways, we assessed the cellular distribution of HDAC2 and NRF2 using immunofluorescence co-staining. The results indicated that HDAC2 and NRF2 proteins were highly co-expressed in the mPFC of sleep deprivation rats. In the Sham group, NRF2 protein was primarily localized to the cytoplasm with minimal co-localization with HDAC2. In contrast, in the SD group, aggregation of NRF2 toward the nucleus and increased co-localization with HDAC2 were observed in the mPFC after 6 days of sleep deprivation [Fig. 5]. We propose that the downregulation of NRF2 protein levels following sleep deprivation and the subsequent changes in oxidative stress levels may be modulated by HDAC2.

### 3.4 HDAC2 regulates pain sensitivity and oxidative stress in the mPFC of sleep deprivation rats by affecting NRF2

**Fig. 6:**
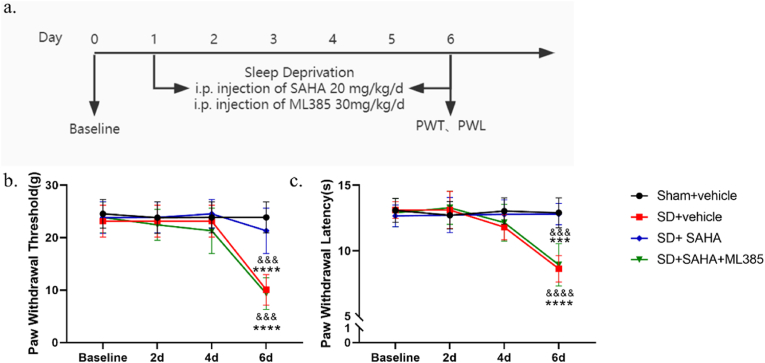
Intraperitoneal injection of SAHA and ML385 reverses the protective effect of SAHA on nociceptive hypersensitivity in sleep deprivation rats. (b,c) Intraperitoneal injection of SAHA and ML385 reversed the protective effect of SAHA on pain sensitivity in sleep deprivation rats (*n* = 8, *∗∗∗p* < 0.001, *∗∗∗∗p* < 0.0001 *vs. Sham + vehicle*; ^*&&&*^*p* < 0.001, ^*&&&&*^*p* < 0.0001 *vs. SD + SAHA*).

**Fig. 7:**
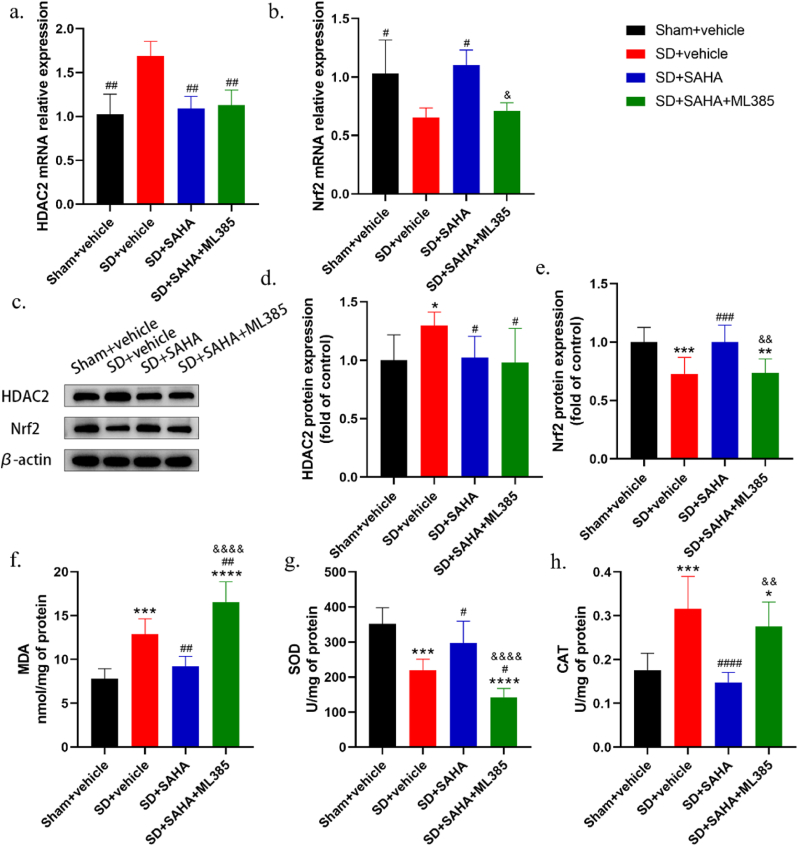
Effects of intraperitoneal injection of SAHA and ML385 on the expression of HDAC2 and NRF2 in the mPFC of sleep deprivation rats. (a, b) qRT-PCR results showed that NRF2 mRNA expression was decreased in the mPFC of rats in the SD + SAHA + ML385 group compared with the SD + SAHA group. NRF2 mRNA expression had no significant difference between the Sham + vehicle group and the SD + SAHA group; there was also no significant difference between the SD + vehicle group and the SD + SAHA + ML385 group (*n* = 4, ^*#*^*p* < 0.05, ^*##*^*p* < 0.01 *vs. SD + vehicle*; ^*&*^*p* < 0.05 *vs. SD + SAHA*). (c–e) Western blot results showed that NRF2 protein expression was decreased in the SD + SAHA + ML385 group compared to the SD + SAHA group. There was no significant difference in NRF2 protein expression between the Sham + vehicle group and the SD + SAHA group; and similarly, there was no difference in NRF2 protein expression between the SD + vehicle group and the SD + SAHA + ML385 group (*n* = 4, *∗p* < 0.05, *∗∗p* < 0.01, *∗∗∗p* < 0.001 *vs. Sham + vehicle*; ^*#*^*p* < 0.05, ^*###*^*p* < 0.001 *vs. SD + vehicle*; ^*&&*^*p* < 0.01 *vs. SD + SAHA*). (f–h) Changes in MDA levels, SOD and CAT activities in the mPFC after intraperitoneal injection of SAHA and ML385 in sleep deprivation rats (*n* = 6, *∗p* < 0.05, *∗∗∗p* < 0.001, *∗∗∗∗p* < 0.0001 *vs. Sham + vehicle*; ^*#*^*p* < 0.05, ^*##*^*p* < 0.01, ^*####*^*p* < 0.0001 *vs. SD + vehicle*; ^*&&*^^*p*^ < 0.01, ^*&&&&*^^*p*^ < 0.0001 *vs. SD + SAHA*).

To further confirm whether inhibiting NRF2 could reverse the protective effects of SAHA on nociceptive hypersensitivity in sleep deprivation rats, ML385, an NRF2 inhibitor, was administered [Fig. 6a]. Compared to the SD + SAHA group, intraperitoneal injection of ML385 significantly reduced PWTs and PWLs [Fig. 6b and c]. Western blot and qPCR experiments revealed that the expression levels of NRF2 were significantly lower in the SD + SAHA + ML385 group compared to both the Sham + vehicle and SD + SAHA groups [Fig. 7a–e].

We assessed the oxidative stress levels in the mPFC of these four groups of rats. The results indicated that SOD activity was significantly reduced in the SD + SAHA + ML385 group compared to the Sham + vehicle and SD + SAHA groups [Fig. 7g], while MDA levels and CAT activity were significantly higher in the same group [Fig. 7f and h]. These findings suggest that inhibiting NRF2 expression with intraperitoneal ML385 reversed the protective effects of SAHA on the oxidative stress status in the mPFC tissue of sleep deprivation rats. There was no significant change in SOD activity, MDA levels, or CAT activity between the SD + SAHA + ML385 group and the SD + vehicle group. The lack of significant difference may be due to sleep deprivation causing oxidative stress damage in the mPFC tissues of rats; however, co-administration of SAHA and ML385 reversed SAHA's protective effects, leading to an oxidative stress state. This suggests that NRF2 is implicated in HDAC2's regulation of nociceptive hypersensitivity and oxidative stress in sleep deprivation rats.

### 3.5 Sleep deprivation influences apoptosis through the HDAC2-NRF2 pathway, as identified by the TUNEL assay

**Fig. 8:**
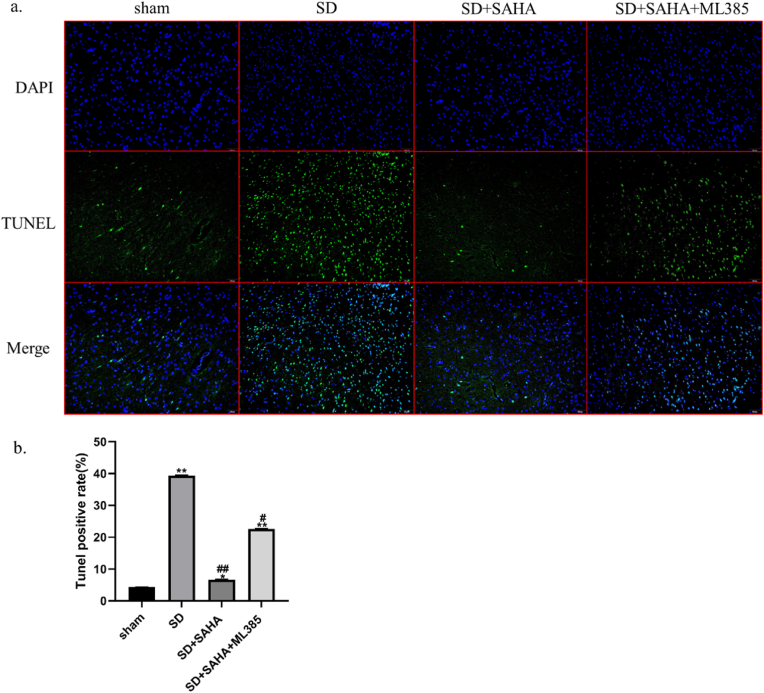
SAHA abolished sleep deprivation induced pyroptosis, and ML385 antagonized the protective effect of SAHA on cells in the mPFC tissue. (a) Pyroptosis was assessed by TUNEL assay. (b) TUNEL positive cells were quantified. (*∗p* < 0.05, *∗∗p*< 0.01 *vs. Sham* ; ^*#*^*p* < 0.05, ^*##*^*p* < 0.01 *vs. SD* ).

In this study, a TdT-mediated dUTP nick end labeling (TUNEL) assay was utilized to ascertain whether sleep deprivation induces neuronal cell apoptosis in the mPFC of rats [Fig. 8]. A significant increase in apoptosis was observed in the mPFC of rats in the SD and SD + SAHA + ML385 groups compared to the Sham group. The rate of apoptosis in the mPFC of rats in the SD + SAHA group was significantly lower than in the SD group, suggesting that intraperitoneal injection of SAHA could counteract sleep deprivation-induced apoptosis in the mPFC of rats. Furthermore, the number of apoptotic cells in the mPFC of the SD + SAHA + ML385 group was significantly higher compared to the SD + SAHA group, suggesting that SAHA's neuroprotective effect in the mPFC may be mediated by restoring NRF2 levels in sleep deprivation rats.

## 4 Discussion

This study aimed to investigate the molecular mechanisms underlying sleep deprivation-induced pain. Our findings indicated a significant increase in HDAC2 expression and a decrease in NRF2 levels in the mPFC of sleep deprivation rats. Treatment with the HDAC inhibitor SAHA effectively alleviated sleep deprivation-induced nociceptive hypersensitivity, normalized HDAC2 expression, and protected the mPFC from oxidative stress and apoptosis. Moreover, SAHA administration significantly enhanced NRF2 expression in the mPFC of sleep deprivation rats, reducing oxidative damage and apoptosis. This points to a possible mechanism by which SAHA exerts its analgesic effect on sleep deprivation-induced nociceptive hypersensitivity. Notably, the NRF2 inhibitor ML385 negated SAHA's protective effects against nociceptive hypersensitivity and oxidative stress induced by sleep deprivation. Sleep deprivation-induced alterations in HDAC2 expression, downregulation of NRF2, and the resultant increase in oxidative stress and apoptosis are implicated as potential drivers of heightened pain sensitivity in rats.

Sleep is a fundamental restorative process for all body systems. While numerous studies have examined the relationship between sleep deprivation and pain behavior, the precise mechanisms through which sleep deprivation results in nociceptive hypersensitivity remain unclear. The acetylation of lysine residues on histones and other proteins, such as transcription factors, is a critical mechanism for regulating gene expression [30]. Histone acetyltransferases (HATs) and HDACs are enzyme groups that regulate the acetylation/deacetylation balance. HDACs have been linked to peripheral sensitization in neuropathic and inflammatory pain [31,32]. Prior evidence indicates that neuropathic pain can cause sustained increases in HDAC2 expression levels. Consequently, to verify the involvement of HDAC2 in sleep deprivation-induced central sensitization, we initially assessed the expression levels of HDAC1 and HDAC2 in the mPFC following sleep deprivation. Our findings showed a significant increase in HDAC2 mRNA and protein expression in the mPFC of rats six days after sleep deprivation, whereas HDAC1 levels remained unchanged. Immunofluorescence double staining demonstrated that HDAC2 expression was predominantly localized to neurons within the mPFC.

There is growing evidence that HDACs play a crucial role in pain regulation. Previous reports have indicated that the HDAC inhibitor SAHA alleviates bone cancer pain and inflammatory pain by upregulating histone acetylation [5,28]. However, the effect of SAHA on ameliorating nociceptive hyperalgesia in sleep deprivation rats has not been clearly demonstrated. Since SAHA has been shown to ameliorate memory deficits in sleep deprivation rats [33], we hypothesized that SAHA might inhibit sleep deprivation-induced nociceptive hyperalgesia by upregulating histone acetylation. Our current study supports this hypothesis, demonstrating that SAHA treatment can ameliorate sleep deprivation-induced behavioral abnormalities. We also discovered that the molecular mechanism by which SAHA affects sleep deprivation may involve the regulation of HDAC2 and NRF2. Moreover, sleep deprivation can cause an imbalance in HAT/HDAC activity and induce oxidative stress [34]. Our study showed that SAHA influences HDAC2 and NRF2 levels, as well as oxidative stress parameters, in the mPFC of sleep deprivation rats. Thus, the present study suggests that SAHA may be a promising candidate for preventing sleep deprivation-induced nociceptive hypersensitivity. While previous studies have demonstrated SAHA's efficacy in bone cancer and inflammatory pain, our research is among the first to reveal its potential in alleviating hyperalgesic behavior in sleep deprivation rats. The positive effects of SAHA on sleep deprivation-induced behavioral abnormalities appear to be associated with its regulation of HDAC2 and NRF2.

Oxidative stress is frequently associated with apoptosis. Sleep deprivation has been shown to induce oxidative stress via activation of the NF-κB pathway, which can trigger neuroinflammation in the brain. This inflammation can subsequently lead to autophagy and apoptosis of neuronal cells, ultimately causing neuronal loss [24]. A study on chronic pain in sleep deprivation rats reported a significant increase in the number of Iba1^+^ and TLR4^+^ cells, along with exacerbated neuronal apoptosis [25]. In line with these results, our study indicates that sleep deprivation can induce oxidative stress and apoptosis, potentially through the interaction between HDAC2 and NRF2. Sleep deprivation elevates HDAC2 expression, which in turn inhibits NRF2 expression, affecting downstream redox reactions and causing a state of oxidative stress in the organism. These changes can lead to neuronal apoptosis, resulting in neuronal loss and, consequently, pain.

The HDAC inhibitor trigonellin-A has been shown to protect neurons both in vitro and in vivo under conditions of glutathione (GSH)-depletion-induced oxidative stress [35]. Valproic acid, another HDAC inhibitor, protects neurons in culture from glutamate-induced excitotoxicity [36]. In our study, SAHA significantly alleviated nociceptive hyperalgesia and oxidative stress in the mPFC of rats following 6 days of sleep deprivation. Our findings indicate that the reduction in oxidative stress damage may be associated with SAHA's protective effect against sleep deprivation-induced nociceptive hypersensitivity in rats. While SAHA has been reported to have analgesic effects [5,28], no studies have explored its impact on sleep deprivation-induced nociceptive hypersensitivity through the HDAC2 and NRF2 pathways. NRF2 regulates the antioxidant system and is activated in response to oxidative stress. Upon activation, NRF2 translocates to the nucleus and interacts with specific DNA sequences known as antioxidant response elements (ARE) [37]. These ARE sites are located in the promoter regions of genes encoding phase II antioxidant proteins. Animals with NRF2 knockout are more susceptible to oxidative stress, and their microglia exhibit heightened inflammation [38,39]. Research has indicated that NRF2's activity is influenced by acetylation, and macrophage-derived extracellular vesicles have been shown to alleviate inflammatory pain by modulating the HDAC2/NRF2 axis [7]. Our study suggests that SAHA mitigates sleep deprivation-induced pain by regulating HDAC2 and NRF2-mediated oxidative stress responses.

The study illuminates the complex interactions among HDACs, NRF2, oxidative stress, and apoptosis in the context of sleep deprivation-induced pain. Increased HDAC2 expression during sleep deprivation suppresses Nrf2 expression, leading to oxidative stress, apoptosis, and heightened pain sensitivity. Our findings support the hypothesis that SAHA's therapeutic effects on sleep deprivation-induced pain are due to its modulation of HDAC2 and NRF2 levels, as well as its regulation of oxidative stress in the mPFC.

In summary, our research indicates that sleep deprivation triggers oxidative stress and apoptosis, which in turn causes pain by increasing HDAC2 and decreasing NRF2. SAHA mitigates these effects by reducing oxidative stress and activating NRF2, suggesting its potential as a treatment for sleep deprivation-induced pain, particularly in individuals with concurrent pain and sleep disorders.

## Assistance with the article

None declared.

## Financial support and sponsorship

This work was supported by Henan Province science and technology research and development projects (No.222102310072); 10.13039/501100001809National Natural Science Foundation of China (82001187); 10.13039/501100001809National Natural Science Foundation of China (82002086). National Natural Science Foundation of China (82101298).

## Conflicts of interest

None declared.
